# The Polarization of Clinician and Service Staff Perspectives After the Use of Health Information Technology in Youth Mental Health Services: Implementation and Evaluation Study

**DOI:** 10.2196/42993

**Published:** 2023-07-25

**Authors:** Sarah McKenna, Sarah Piper, William Capon, Alison Crowley, Lucas Lira, Haley M LaMonica, Min Kyung Chong, Elizabeth Scott, Ian Hickie, Frank Iorfino

**Affiliations:** 1 Faculty of Medicine and Health Brain and Mind Centre The University of Sydney Camperdown Australia; 2 Mind Plasticity Sydney Australia

**Keywords:** mental health, youth, adolescent, service delivery, implementation science, digital technologies, measurement-based care, health information technology, information system, perspective, provider, health care staff, health care worker, health care professional

## Abstract

**Background:**

Highly personalized care is substantially improved by technology platforms that assess and track patient outcomes. However, evidence regarding how to successfully implement technology in real-world mental health settings is limited.

**Objective:**

This study aimed to naturalistically monitor how a health information technology (HIT) platform was used within 2 real-world mental health service settings to gain practical insights into how HIT can be implemented and sustained to improve mental health service delivery.

**Methods:**

An HIT (The Innowell Platform) was naturally implemented in 2 youth mental health services in Sydney, Australia. Web-based surveys (n=19) and implementation logs were used to investigate staff attitudes toward technology before and after implementation. Descriptive statistics were used to track staff attitudes over time, whereas qualitative thematic analysis was used to explore implementation log data to gain practical insights into useful implementation strategies in real-world settings.

**Results:**

After the implementation, the staff were nearly 3 times more likely to agree that the HIT would *improve care for their clients* (3/12, 25% agreed before the implementation compared with 7/10, 70% after the implementation). Despite this, there was also an increase in the number of staff who disagreed that the HIT would improve care (from 1/12, 8% to 2/10, 20%). There was also decreased uncertainty (from 6/12, 50% to 3/10, 30%) about the willingness of the service to *implement the technology for its intended purpose*, with similar increases in the number of staff who agreed and disagreed with this statement. Staff were more likely to be uncertain about whether *colleagues in my service are receptive to changes in clinical processes* (*not sure* rose from 5/12, 42% to 7/10, 70%). They were also more likely to report that their service *already provides the best mental health care* (agreement rose from 7/12, 58% to 8/10, 80%). After the implementation, a greater proportion of participants reported that the HIT enabled shared or collaborative decision-making with young people (2/10, 20%, compared with 1/12, 8%), enabled clients to proactively work on their mental health care through digital technologies (3/10, 30%, compared with 2/12, 16%), and improved their response to suicidal risk (4/10, 40% compared with 3/12, 25%).

**Conclusions:**

This study raises important questions about why clinicians, who have the same training and support in using technology, develop more polarized opinions on its usefulness after implementation. It seems that the uptake of HIT is heavily influenced by a clinician’s underlying beliefs and attitudes toward clinical practice in general as well as the role of technology, rather than their knowledge or the ease of use of the HIT in question.

## Introduction

### Background

The development of health information technologies (HITs) has seen recent and rapid expansion to address the well-established shortcomings within the mental health system [1-3]. In Australia and globally, widespread issues persist across the mental health system at both a structural level (ie, the arrangement and operation of services) and clinical level (ie, how care is delivered to individuals), which impact the outcomes of individuals seeking mental health care [4,5]. Issues include limited access, extensive waitlists, fragmented and disconnected services, and a lack of fundamental clinical practices that ensure that individuals receive personalized care appropriate to their level of need, such as measurement-based routine outcome monitoring and care coordination [6,7]. The COVID-19 pandemic and the resulting limitations of face-to-face care have seen a further push to implement HITs within mental health care and an increased need for literature to guide this [8,9].

More specifically, there is a call for youth mental health services to implement technologies that can facilitate more personalized care through detailed assessment and tracking of multidimensional outcomes and efficient multidisciplinary care coordination [10,11]. In Australia’s most recent study of mental health and well-being, almost half (46.6%) of female individuals aged 16 to 24 years and almost one-third (31.2%) of male individuals aged 16 to 24 years had experienced symptoms of a mental disorder in the past 12 months, which is far higher than any other age group, making youth mental health care an urgent priority [12]. A primary solution has been the funding of *headspace*, the National Youth Mental Health Foundation, which is mandated to establish youth-friendly, highly accessible centers that provide multidisciplinary enhanced primary care [13-15]. However, longitudinal and large cohort studies of youth accessing these services have found that only a small proportion experienced significant improvement in mental health or psychosocial functioning [16,17]. Possible explanations for this include limited resources and lack of qualified staff, particularly in rural areas, limiting the capacity of services to identify and respond to emerging mental disorders early and appropriately [4,16]. Thus, youth mental health services should be better equipped to triage care options based on levels of need (such as group therapy for clients who are at a low risk and individual therapy for clients who are at a higher risk) and to address the complexity of young people’s needs through multidisciplinary care options [4,11].

### The Need for Technology-Enabled Monitoring and Care

Reviews have suggested that technology-enabled routine outcome monitoring leads to improved outcomes and reduced dropout rates from mental health care systems [18-20]. These effects are particularly strong for clients who are *not on track*, likely because outcome monitoring enables clinicians and clients to compare treatment progress with goals more easily and adjust therapy as needed [19]. Accordingly, the Australian Productivity Commission strongly recommended that mental health services improve their ability to *provide the right health care at the right time for those with mental illness*, specifically emphasizing that *technology should play a larger role by improving assessment and referrals* [10]. Thus, there is a strong impetus for youth mental health services to implement technology platforms that can improve the personalization of care for young people.

There are few studies and sparse literature to guide the implementation of HITs within mental health care services and to detail how they can be best used and sustained within a variety of service settings. Recent reviews of existing literature on HIT have found that user engagement is a consistent problem, varies from study to study, and is generally lower in real-world settings than in research studies [3,21,22]. For example, participant adherence to internet-based cognitive behavioral therapy can range from 6% to 100% [23]. Moreover, the implementation literature typically focuses on individual uptake, whereas there is a need to address the implementation of HIT at a service level to achieve systemic improvements in assessment, triaging, and care coordination. Some existing research suggests that the uptake of HIT by mental health professionals is commonly limited by poor digital literacy, concerns about time or financial burdens, and lack of support from service leadership [19,20,24]. However, a review of 208 articles on digital mental health interventions found only 14 articles that included a description of implementation strategies and therefore could be used to inform future HIT implementation [22]. Taken together, a stronger evidence base from real-world settings is needed to guide the successful implementation of HIT in youth mental health services.

### The Development of an HIT (The Innowell Platform)

**Figure 1:**
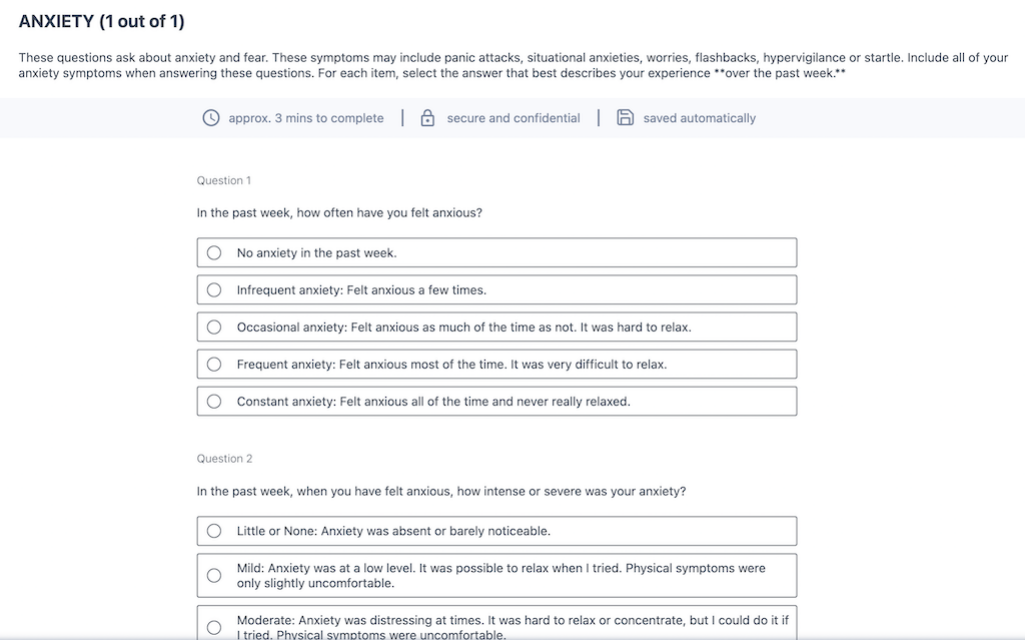
Example of the Anxiety question set within the web-based questionnaire and example of the dashboard of results from the web-based questionnaire.

**Figure 2:**
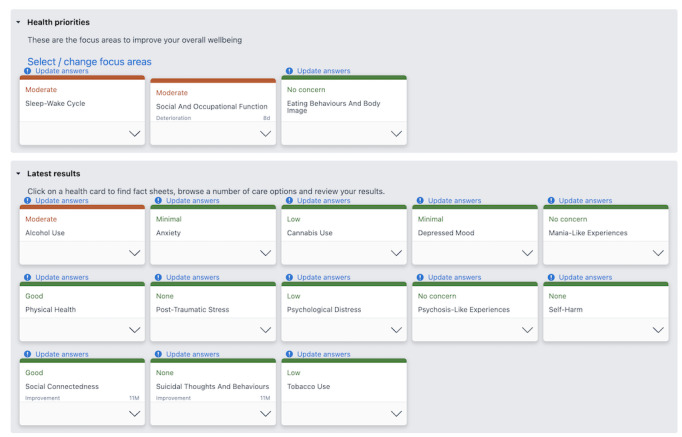
Example of the dashboard of results from the web-based questionnaire.

The University of Sydney’s Brain and Mind Centre (the Youth Mental Health and Technology team) has developed an HIT in partnership with young people with lived experience of mental illness, their families, clinicians, and service administrators [11]. The Brain and Mind Centre Youth Model of Care underpins this solution, arguing that multidisciplinary assessment and continuous monitoring should be used to identify the underlying trajectories of mental disorders and accurately assign the different types and levels of care according to individual needs [11]. To facilitate these clinical processes, the Innowell Platform was designed as a joint partnership between the University of Sydney, PwC (Australia), and Innowell to facilitate measurement-based mental health care [25-27] by collecting, tracking, and reporting health information back to the individual and their clinicians to inform collaborative decision-making and personalized care [28,29]. Textbox 1 provides a description of the functionalities of the technology. Notably, both the individual and clinician can access the individual’s health information, promoting transparency of care; this is explained in detail to the client when they are invited to use Innowell. Figure 1 provides an example of the web-based questionnaire completed by the individual, and Figure 2 shows the dashboard of results available to the clinician and client.

Innowell was co-designed and implemented in various youth mental health services through Project Synergy, as has been described in detail in previous publications [25-27]. A core feature of the implementation process was co-designing implementation strategies with services through an iterative process that allowed the research team to reflexively adapt to the individual services to address unique challenges that may be present in each setting. Previous studies have outlined the framework that was used to inform this co-design process; however, there is a need to further investigate how the co-designed strategies operated within the *real-world* service settings and how suitable these strategies were once implemented. Accordingly, this paper describes a preliminary observational analysis of real-world HIT implementation.

**Description of the functionalities of the health information technology (The Innowell Platform).**

- Multidimensional assessment across a range of biopsychosocial domains (eg, depressed mood, physical health, and sleep)
- Identification of suicidal thoughts and behaviors and subsequent notification to treating clinician and service
- Immediate dashboard of results across the range of biopsychosocial domains (as collected via the multidimensional assessment)
- Algorithms to determine the severity of needs across these biopsychosocial domains
- Data tracking and web-based progress report
- Optional support person input and health information sharing
- Health priority setting whereby people can identify 3 domains of mental health and well-being they would like to work on
- Coordination of care across multidisciplinary services
- Multiple user roles tailored to clinicians, service administrators, and individuals seeking care

### Aims

This study aimed to monitor and evaluate how the HIT (The Innowell Platform) was used naturalistically within 2 mental health services to gain practical insights into how an HIT can be best implemented and sustained to improve mental health service delivery. Furthermore, this study aimed to investigate the digital readiness of mental health service staff, the use of common clinical practices, and whether these practices can be enhanced using an HIT.

## Methods

### Study Design

A prospective study design was used, which included the implementation of the HIT in 2 participating sites. Data were collected via web-based surveys (at a 3-month interval over a 12-month period) and implementation logs (fortnightly) to explore clinical and service perspectives on how the HIT could be best used to facilitate improved clinical processes and outcomes within the service and to measure attitudes around the use of digital technologies in mental health care.

### Implementation of the HIT

The HIT was implemented in 2 participating mental health services for 12 months (both sites chose to extend the implementation without the accompanying research measures after this period). The sites included *headspace* Camperdown and Mind Plasticity. *Headspace* Camperdown is a Commonwealth government–funded, youth-friendly, and multidisciplinary service offering early-intervention mental and physical health care and vocational support to young people aged 12 to 25 years [13]. The service has 21 staff members and is located within inner-city Sydney and provides care to approximately 1200 young people per year via psychology, psychiatry, occupational therapy, general practice, and exercise physiology. Mind Plasticity, a private, specialist practice consortia, offers multidisciplinary care to individuals of all ages who require mental health support. The service is also based in inner-city Sydney and consists of 22 staff offering psychology, psychiatry, and occupational therapy as well as education support, speech pathology, and neuropsychology services. Both sites also have a mix of contractors and employed staff.

Implementation was guided by a strategy for implementation science [26], which was developed and tested through a series of Australian government–funded research studies that implemented an HIT across a range of Australian mental health services with the aim of transforming the way mental health services deliver care to individuals [26,27,29]. Implementation phases include scoping and feasibility (assessing service resources and readiness including staffing capacity and IT requirements) and co-designing and configuring the HIT content to suit the needs of the services (eg, ensuring care options offered in the HIT reflect what the services offer, reviewing suicide notification functionality, and offering education and training on the HIT).

Implementation strategies were standardized across both settings; although once implemented, the services established their own methods of using the HIT within their service, both administratively and clinically. For example, *headspace* Camperdown offered the HIT’s web-based questionnaire to new clients before their first face-to-face appointment with a clinician, using this feature primarily for initial assessment, whereas Mind Plasticity offered the HIT’s web-based questionnaire to existing clients of the practice, primarily for the purpose of routine outcome monitoring. This naturalistic approach allowed researchers to observe the impact of the HIT and collect data from service staff regarding how best to use the HIT under ecologically valid conditions that reflected a *real-world* service setting.

### Recruitment and Informed Consent

All service staff, including clinicians, service managers, and service administrators, were invited to participate in this study. The participation of a broad range of service staff ensured that the feedback was collected at multiple levels for each service, including both administrative and clinical stakeholders. Eligible staff were invited to participate in web-based surveys via email from a member of the research team. If the staff indicated an interest in participating, they would receive a participant information and consent form and a survey link to provide their nonidentifiable data.

### Participant Inclusion Criteria

Potential participants were required to meet the below inclusion criteria to participate in this study.

- Current staff (eg, clinicians, service managers, or administrators) who work at a participating mental health service
- Aged ≥18 years
- English proficiency
- Completion of the required consent processes

### Evaluation of Clinical Opinions and the HIT

#### Web-Based Surveys

Web-based surveys were administered to the participants (clinicians, service managers, and service administrators) using the electronic data collection software REDCap (Research Electronic Data Capture; Vanderbilt University) [30]. The surveys were based on our team’s previous research evaluating the impact of HITs on mental health services across Australia [31], with survey questions adapted and added to address the aims of this study. Specifically, data were collected about current clinical practices; if the HIT supported clinical practices; and beliefs and attitudes toward the adoption of HITs within the service, including digital readiness of staff, barriers and facilitators to adoption, and feedback on outcomes (positive or negative) that resulted from the implementation of the technology.

Participants were invited to complete a *baseline* survey before or during the initial phases of HIT implementation. After the completion of the baseline survey, follow-up surveys were distributed to participants at 3-month intervals to compare the effect of implementing the HIT on clinical practice over 12 months. Owing to low uptake, we were only able to report the findings from the 12-month follow-up. Multimedia Appendix 1 provides a copy of the baseline web-based survey.

#### Implementation Logs

Implementation logs were completed monthly in REDCap by an implementation officer, who was a member of the research team and whose role included supporting the implementation of the HIT within the participating services (eg, providing educational resources, supporting the onboarding of staff to the technology, and facilitating technical support), distributing web-based surveys to staff, and collating feedback from service staff regarding the digital health technology. The implementation logs comprised questions adapted from the Quality Implementation Framework [32] and allowed us to naturalistically evaluate the extent to which implementation processes aligned with the best practice and to document the barriers or facilitators of HIT uptake. The logs were used to document observations made by the implementation officer, over the course of a year, based on fortnightly summaries of meetings; interactions; and emails from the service staff about critical steps in implementation, such as what changes were undertaken by the service to best use the technology (eg, service pathway changes and changes in staffing or staff roles), any technical modifications required of the HIT to improve its utility, and what aspects of the HIT and its implementation have been effective or ineffective within the service (refer to Multimedia Appendix 2 for an example of the implementation logs). Importantly, the implementation officer aimed to embed themselves within the service where possible, primarily through the attendance of service staff meetings, to ensure that the observations from the implementation of the HIT were collected from within the service, with minimal disturbance, under *real-world* conditions. Table 1 provides further details on the methods by which observations were naturalistically collected by the implementation officer to complete the implementation logs.

**Table 1:** Methods used to observe the implementation processes.

Method	Service	Staff involved	Attendance	Details
Case review or intake meeting	*headspace* Camperdown	All clinical staff, service manager, and research officer	Weekly	Meetings involved collaboratively reviewing client progress and triaging recent client intakes. The HIT^a^ was used to display client clinical information for team discussion.
Peer review meeting	Mind Plasticity	All clinical staff, service manager, and research officer	Monthly	Meetings involved discussion and review of client or patient progress and discussion of research projects and other collaborations when relevant (including the implementation of the HIT).
Weekly administration meeting	Mind Plasticity	Practice manager and research officer	Weekly	Meeting involved an update or discussion on the progress of the HIT implementation. This included any new developments within the service, issues or challenges, questions, or feedback from staff using the HIT.
Email correspondence and other interactions	*headspace* Camperdown and Mind Plasticity	All service staff	When required	All service staff were provided with the research officer’s contact details and were encouraged to contact them with any questions or feedback regarding the implementation of the HIT.

^a^HIT: health information technology.

#### Data Analysis

We used web-based surveys to collect quantitative data on staff attitudes and HIT uptake. We used descriptive statistics to compare responses before and after the implementation. Given the small sample size, it was not possible to analyze the significance of this change through quantitative methods. Qualitative data captured via implementation logs were analyzed using thematic analysis techniques and a constructivist grounded theory approach [33,34], with the aim of establishing themes regarding the use and implementation of the digital health technology within the service. An implementation officer who had been embedded in both health services established an initial list of codes based on data collected from the implementation logs. This analysis focused on identifying the barriers and facilitators of HIT uptake. Subsequently, these codes were shared and discussed with an independent researcher in a face-to-face meeting, and a list of themes was established. Subsequently, the implementation officer conducted a second round of coding to establish broader patterns of meaning within each theme. The themes were again shared with the independent researcher and refined during a face-to-face meeting. A constant comparison of similarities and differences between themes was used to identify the links between themes and to condense the overlapping themes.

Our qualitative data analysis followed the constructivist grounded theory, which assumes that all knowledge is constructed by the meanings that individuals bring to data analysis [35]. As a multidisciplinary team, our existing practical and theoretical perspectives shaped the organization of data into themes; understanding these perspectives can help explain how our sensitivities shaped our interpretation of the implementation process. The primary coder (SP) is an implementation officer who has experience working alongside youth mental health services in Australia to enhance the uptake of HITs and has a strong understanding of implementation science. The secondary coder (SM) was a clinical psychologist and academic researcher experienced in working with young people in a clinical role in youth mental health settings. Implementation science emphasizes the systemic processes that facilitate or limit the use of technology platforms in health settings. Psychological perspectives emphasize that organizational processes are underpinned by interpersonal dynamics linked to the cognitions, attitudes, and beliefs of staff within the service. Again, these perspectives informed the organization of the data into themes.

### Ethics Approval

Ethics approval was obtained from the Executive Ethical Review Panel of the Sydney Local Health District Human Research Ethics Committee, Concord Repatriation General Hospital (2019/ETH13172). Site-specific approval was obtained for *headspace* Camperdown and Mind Plasticity from The University of Sydney and Sydney Local Health District, respectively.

## Results

### Participants and Settings

Across the 2 participating services, 43 individuals were invited to participate in this study. Of the 43 participants, 19 (44%) consented to participate in the study and completed at least 1 web-based survey. A total 63% (12/19) female and 37% (7/19) male participants, who worked across a diverse range of disciplines, were included in this study. Table 2 presents an overview of the participants’ disciplines across participating services. Owing to limited uptake from *headspace* Camperdown, the results were analyzed and presented using data from both services combined.

**Table 2:** Participants’ disciplines across participating services^a^.

Role or discipline^a,b^	Service	
	Mind Plasticity (n=16), n (%)	*headspace* Camperdown (n=3), n (%)
Clinical psychologist	2 (13)	1 (33)
General psychologist	4 (25)	N/A^c^
Provisional psychologist	1 (6)	N/A
Psychiatrist	3 (19)	N/A
Occupational therapist	2 (13)	N/A
Youth access clinician	N/A	2 (67)
Allied health assistant	1 (6)	N/A
General practitioner	1 (6)	N/A
Mental health nurse	1 (6)	N/A
Service administrator	3 (19)	N/A

^a^Please note that 2 participants held a dual role within the service (eg, clinical psychologist and service administrator), resulting in 21 participants.

^b^The mean number of years spent in each role was 7.0 (SD 9.0) years.

^c^N/A: not applicable.

### Staff Beliefs, Attitudes, and Uptake of the HIT

**Figure 3:**
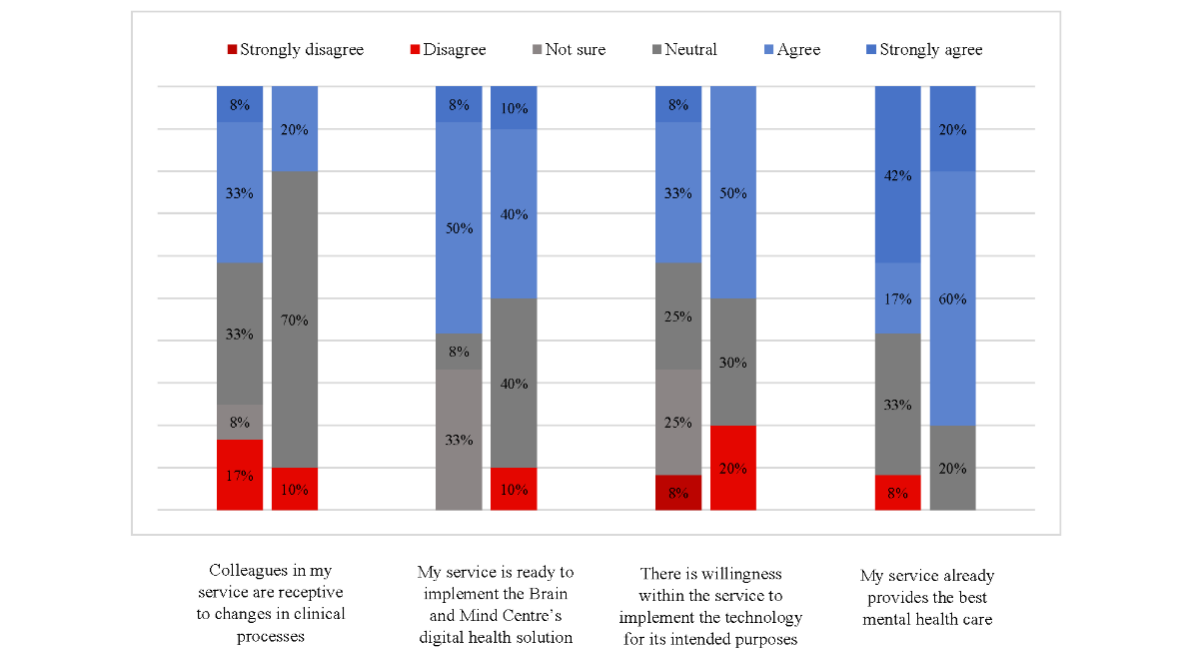
Staff attitudes and beliefs toward the health information technology in service.

**Figure 4:**
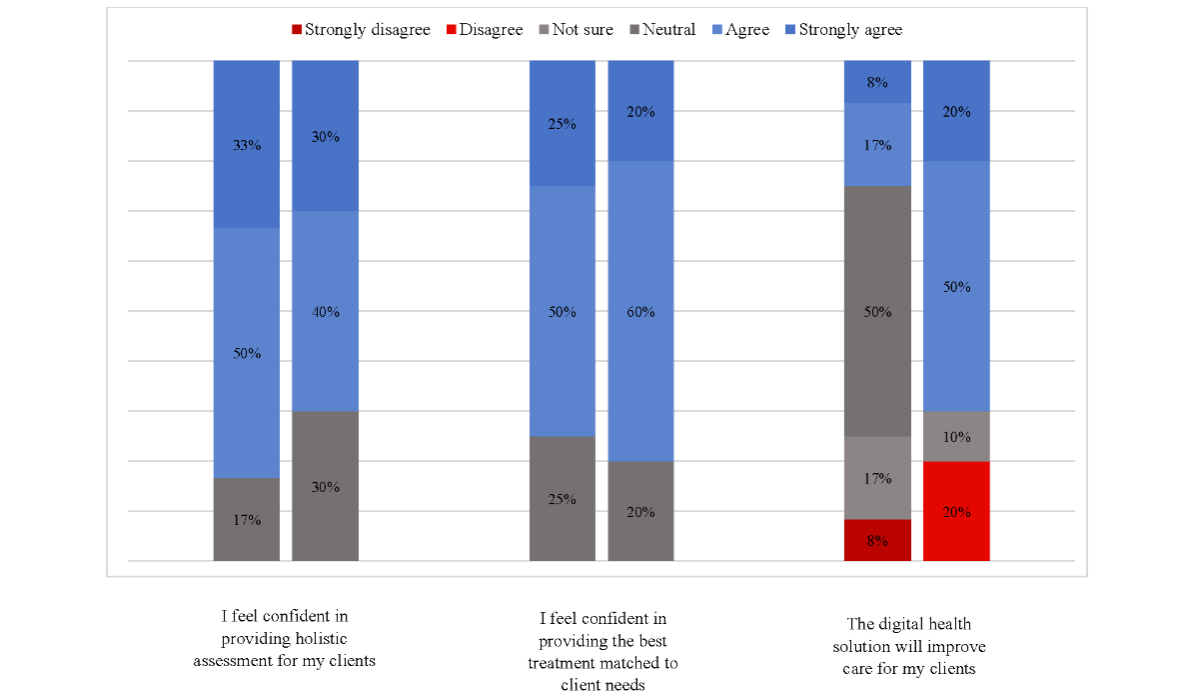
Staff attitudes and beliefs toward the health information technology for individual practice.

Figures 3 and 4 display staff attitudes toward the HIT, both before and after implementation. Relative to baseline, staff attitudes toward the HIT became more polarized after the implementation. After the implementation, the staff were nearly 3 times more likely to agree or strongly agree that the HIT would *improve care for their clients* (3/12, 25% agreed or strongly agreed before the implementation compared with 7/10, 70% after the implementation; Figure 2). Despite this, there was also an increase in the number of staff who disagreed that the HIT would improve care (from 1/12, 8% to 2/10, 20%). There was also decreased uncertainty (from 6/12, 50% to 3/10, 30% who selected *not sure* or *neutral*) about the willingness of the service to *implement the technology for its intended purpose* and similar rate of increase in the number of staff who agreed and disagreed with this statement.

Simultaneously, observing the implementation of new technology in their service changed the staffs’ attitudes toward their colleagues’ clinical practice. Staff were more likely to be uncertain about whether *colleagues in my service are receptive to changes in clinical processes* (the percentage of staff who were *not sure* or *neutral* rose from 5/12, 42% to 7/10, 70%). They were also more likely to report that their service *already provides the best mental health care* (agreement and strong agreement rose from 7/12, 58% to 8/10, 80%). Regarding how the platform was being used, after the implementation, a greater proportion of participants *agree* or *strongly agree* that the HIT enabled shared or collaborative decision-making with young people under their care (2/10, 20%, compared with 1/12, 8%) and enabled clients to proactively work on their mental health care through digital technologies (3/10, 30%, compared with 2/12, 16%); including apps and e-tools other than Innowell). A greater proportion of staff also *agree* or *strongly agree* that the HIT improved their assessment of and response to suicidal risk (4/10, 40% postimplementation, compared with 3/12, 25% preimplementation).

Multimedia Appendix 3, Implementation themes and associated mitigation strategies, displays the themes extracted from the implementation logs and the associated mitigation strategies adopted by the researchers and service staff.

## Discussion

### Principal Findings

This study assessed the perspectives of mental health service staff on an HIT platform during and after implementation and aimed to observe and evaluate the effect of the implementation process on clinical practices. Implementation log data revealed various strategies that were used by these services to support technology implementation, including education and training, *on-the-ground* administrative support, staggered implementation, use in team meetings, and continuous feedback to technology developers. However, despite exposure to similar implementation strategies, we found that staff attitudes toward the technology became polarized over time, both in terms of their willingness to use the platform and their belief that others in the service would be willing to adopt HIT. Thus, it appears that implementation approaches may need to be highly individualized to clinicians, and strong leadership from service managers and funders is needed to support the successful uptake of HIT.

Given that clinicians were exposed to the same technology and implementation strategies yet had polarized reactions to the technology, the uptake of HIT in health services may ultimately be severely influenced by factors unrelated to the HIT or implementation approach. A potential explanation derived from the current literature may be that the uptake of HIT is linked to a clinician’s existing beliefs and attitudes toward clinical practice and technology, over and above their knowledge of or the ease of use of the HIT in question [19,36]. For example, previous research has found that individual processes such as internal feedback propensity, self-efficacy, and commitment to use feedback mitigate the therapist’s use of routine outcome monitoring technology [36]. In addition, common barriers to HIT implementation are that mental health professionals are often overscheduled, lack time to implement new practices, lack confidence in the confidentiality of the data, and fear that the data will not be interpreted reliably by managers or funders [37]. In summary, future research should explore the extent to which individualizing implementation strategies for health care professionals within services can improve the overall uptake of HIT.

Alternatively, service managers, policy makers, and funders need to explore how clinicians can be supported to engage in new clinical practices and make the best use of new HITs. Previous work has found that introducing new HITs or clinical practices is most likely to be sustained when a “critical mass” of staff routinely implements the new tool in their practice [38]. This allows clinicians to become more comfortable with the HIT or intervention, see it integrated into routine practice, and access peer support for the technical and emotional aspects of implementation. Accordingly, organizational support in the form of service-wide policy change, leadership from managers, and new processes to integrate HIT in clinical practice is needed so that the staff feel positively supported by the service and their colleagues to implement new HITs [38,39].

This study has important implications for policy makers, funders, and implementation science. Services may require much more significant incentives to adapt new processes and pathways that leverage the use of HIT to improve service quality. These incentives may be financial, legal, or regulatory in nature and may also arise opportunistically, for example, when mental health services were forced to adopt telehealth owing to the COVID-19 pandemic [40]. There is strong support from leading organizations such as the Australian Productivity Commission [41] and the Institute of Medicine [42] for the widespread use of HIT in services to provide person-centered and measurement-based care. This needs to be urgently backed up by key policies that provide services with the impetus for change.

### Limitations

Notwithstanding these contributions, our study had some limitations. First, only 14 participants involved in clinical care completed aspects of the web-based survey regarding their attitudes toward using HIT to enhance clinical practice, which reduces the generalizability of our findings. Recruitment issues in eHealth trials have been well documented [43]. Despite the limited sample size, the in-depth evaluation of a real-world clinical service implementing a new digital technology has provided invaluable qualitative data that reflect the real-world challenges of this work. There was low readiness among staff to use HIT; thus, the small sample size in our study may reflect a general reluctance among clinicians to adopt HIT. This creates a further impetus for researchers and clinicians to continue evaluating approaches that can facilitate the implementation and use of HIT in real-world health care settings. In addition, despite identifying various processes that were used in a naturalistic mental health service to facilitate the implementation and use of HIT, our study did not evaluate the effectiveness of these processes. This was because we adopted a prospective study design that aimed to monitor how HITs were used and implemented as well as investigate digital readiness among staff. Future research is needed to evaluate the efficacy of the approaches identified in our study in increasing the implementation and use of HIT. Finally, qualitative data collection involved observations recorded by an implementation officer on implementation logs. This approach was chosen because it allowed us to naturalistically observe barriers and facilitators of HIT implementation at the service level rather than focusing on individual clinicians’ experiences. Even so, this creates a need for future research to more rigorously evaluate the underlying beliefs and attitudes that explain clinicians’ polarized experiences with HIT implementation through qualitative methods such as semistructured interviews.

### Conclusions

Overall, our findings have broader implications for the future implementation of HITs in mental health services. Clinicians exposed to the same HIT, education, and support had polarized attitudes about the use of the technology, suggesting that the uptake was linked to internalized views about clinical practice and technology rather than knowledge of or the ease of use of the platform itself.

## Appendix

Multimedia Appendix 1 Baseline web-based survey.

Multimedia Appendix 2 Implementation log.

Multimedia Appendix 3 Implementation themes and associated mitigation strategies.

## Abbreviations

HIT: health information technology
REDCap: Research Electronic Data Capture

## Data Availability

The data that support the findings of this study are available from the corresponding author upon reasonable request.
