# Supplementary material for: The Polarization of Clinician and Service Staff Perspectives After the Use of Health Information Technology in Youth Mental Health Services: Implementation and Evaluation Study
Source: JMIR Hum Factors. 2023 Jul 25;10:e42993. doi: 10.2196/42993 (PMC10410532; doi:10.2196/42993)
Supplement: Multimedia Appendix 3 [file humanfactors_v10i1e42993_app3.docx]

Multimedia Appendix 3

Implementation themes and associated mitigation strategies.

| Theme | Details | Implementation strategies adopted to address the theme |
| --- | --- | --- |
| Limited staff capacity | - Time constraints and limited staff capacity were present at both participating services, resulting in few opportunities to engage and communicate with staff regarding the use of the HIT^a^ within the service. Limited staff capacity was the result of an increase in client demand and case load, partly driven up by the effects of COVID-19. - “Clinicians are booked from 8am until 6pm without a break, leaving limited opportunity for researchers to engage with clinicians.” [Implementation officer, Mind Plasticity] | - Adaptable and flexible education and training - Reduced opportunities to meet, train, and educate staff in the functional navigation of the HIT and the utility of the HIT within the service resulted in a need for education and training to be flexible to meet the differing needs and constraints of staff. Researchers implemented the following:   - A reduction in the length of training sessions (to prioritize key clinical concepts, eg, risk notifications, dashboard overview, and care options).   - A *train-the trainer* model. A service administrator or clinician was trained by a researcher on the use of the HIT and could be available on site to provide training sessions for staff in line with their availability. - On-the-ground support - Service staff emphasized the need for administrative and clinical support “on the ground” to implement the technology, via a “digital navigator.” A digital navigator was important, given the lack of time capacity available to administrative and clinical staff, particularly in the initial stages of implementing the technology. - The digital navigator role included the following:   - Ensuring that clients/patients were regularly engaging with the HIT. This included sending them reminders (via email) to complete and update their clinical questionnaire within the HIT.   - Supporting and encouraging clinicians to engage with the HIT, including checking client/patient updates within the HIT before face-to-face appointments.   - Providing additional education and training. |
| Trust in technology | - Managerial staff at both services expressed concern about the impact of a new HIT on the service. Concerns included the impact on and *buy in* from clinical staff and how a change to their practice via a new technology would be received (particularly acknowledging limited staff capacity and multiple technology platforms already used within services). - “Staff are already utilising multiple administrative Platforms on a daily basis—a gradual introduction to staff is favoured by the service manager.” [Implementation officer, Mind Plasticity] | - A staggered implementation approach - A small group of key senior clinicians and administrative staff adopted the HIT within their practice, before implementing more widely across the service. This key group of clinical (and administrative) staff were selected by the service to ensure that when a client used the technology, a sufficient range of staff disciplines using the HIT were available to provide the client with the care that they required. - Mind Plasticity: initial implementation included the service manager, a service administrator, a clinical psychologist, an occupational therapist, a mental health nurse, and a psychiatrist. - *Headspace* Camperdown: initial implementation included the service manager and 3 youth access clinicians. - As directed by the management at each service, additional staff were invited to onboard to the HIT over the coming weeks. - Technology used with existing service clients. Key clinical staff who were using the technology as part of their practice invited only their existing clients to use the technology in the early stages of implementation (new clients did not use the HIT). This approach ensured that clinicians mastered the use of the HIT with familiar clients with whom they had a preexisting relationship, and, at their own pace, they were able to introduce their clients to the use of the technology in a supportive environment. |
| Technology to support team-based care | - Services exhibited different levels of *team-based* interaction and collaboration between clinicians regarding client care. *Headspace* Camperdown used team-based care with regard to the young person’s care via intake and case review meetings. Mind Plasticity had limited team collaboration regarding client care, with a tendency for clinicians to work independently and to “stay in their lane” (senior clinician, Mind Plasticity) | - Technology used in team meetings to support team-based care - The HIT was used within clinical review/intake team meetings at *headspace* Camperdown, displaying client clinical information (their dashboard of results) for review and discussion between clinicians to support collaborative team-based care and care coordination. |
| Technology function informed by service use | - Service staff provided feedback regarding potential improvements to the HIT’s functionalities to better support both clinical and administrative practices within the service. | - Continuous feedback loop to improve technology   - A continuous feedback loop between service staff, researchers, and the HIT’s manufacturer (ie, Innowell) was established to improve and further refine the HIT’s functionality, to enhance its ease of use and ensure it suited the broad needs of a mental health service. Service staff provided feedback to a researcher, who then communicated it to Innowell staff, who determined whether an update would be built into the technology. - Example of updates made to the HIT based on *real-world* service feedback included:   - - Refining the HIT’s *filter* functionality to allow clinicians to more easily view which clients/patients had most recently updated their clinical information     - Increasing the automatic log-out functionality to avoid staff continuously logging back in     - Improving the *look and feel* of the dashboard of results to improve usability and accessibility     - Improving the ease of switching roles within the HIT (ie, switching between clinician, service manager, and admin roles) - “Clinicians and administrators have been motivated by the changes implemented into the technology, as they have come directly from their experiences using it within a service setting.” [Implementation officer] |
| ^a^HIT: health information technology. | | |
